# From Transient Knockdown to Density-Driven Collapse: A Mechanistic Comparison of Adult Mosquito Control by Space Spraying and Mass Trapping in Maldivian Islands

**DOI:** 10.3390/insects17050471

**Published:** 2026-05-02

**Authors:** Bart G. J. Knols, Nabeel Siddiqui, Akib Jahir, Martin Geier

**Affiliations:** 1 K&S Holding BV, Kalkestraat 20, 6669 CP Dodewaard, The Netherlands; 2 Ifakara Health Institute, Ifakara P.O. Box 53, Tanzania; 3 Soneva Fushi, 4th Floor Jazeera Building, Boduthakurufaanu Magu, Male 20077, Maldives; nabeel@soneva.com; 4 Department of Biomedical and Life Sciences, Faculty of Health and Medicine, Furness Building, Furness College, Lancaster University, Bailrigg, Lancaster LA1 4YG, UK; a.akibjahir@lancaster.ac.uk; 5 Biogents AG, An der Irler Höhe 3a, 93055 Regensburg, Germany; martin.geier@biogents.com

**Keywords:** mosquito, space spraying, mass trapping, density, population dynamics, islands, Maldives, vector control, arbovirus

## Abstract

Space spraying and mass trapping represent distinct adult mosquito control strategies with fundamentally different population-level consequences. We developed a density-regulated population model incorporating either pulsed mortality from spraying (efficacy α = 0.2–0.6 per application) or continuous proportional removal through trapping. Parameters were derived from empirical island datasets. Spraying produced rapid but transient reductions and required high-frequency re-application (weekly to daily) to approach structural suppression when α ≤ 0.4. Consistent with model predictions, analysis of Maldivian resort practices indicated that daily spraying is common in operational settings. In contrast, trapping shifted long-term equilibrium density and induced population collapse above a model-estimated threshold density that, under the baseline parameterization used here, corresponded to approximately 8–10 traps per hectare in geographically bounded island systems. Sensitivity analyses demonstrated that qualitative outcomes were robust across plausible parameter ranges. Economic comparison indicated that spraying frequencies required to match threshold trapping substantially increased cumulative cost. In small island settings with negligible immigration, threshold-based mass trapping provides a structurally stable pathway toward sustained suppression, whereas spraying remains primarily a rapid-response tool with limited durability under moderate-efficacy conditions. When potential ecological externalities and human health considerations associated with repeated insecticide use are considered, trapping may offer additional advantages beyond purely economic comparisons.

## Simple Summary

Mosquito control in Maldivian resort islands commonly relies on frequent insecticide space spraying to reduce adult populations. However, spraying produces short-term knockdown and often requires repeated applications to maintain suppression. In contrast, mass trapping continuously removes host-seeking females and may alter long-term population dynamics. Using a mechanistic population model calibrated with empirical island data, we compared spraying and trapping under equivalent removal intensity. Our results show that while spraying can provide rapid temporary reductions, sustained suppression requires high application frequency. Mass trapping, when deployed at sufficiently high density, can shift population equilibrium and potentially drive collapse in geographically bounded systems. Economic comparisons indicate that once high-frequency spraying is required, trapping at threshold density can achieve comparable or greater suppression at similar or lower long-term cost. Moreover, when potential ecological externalities and human health considerations associated with repeated insecticide application are considered, mass trapping may offer additional advantages beyond purely economic comparisons.

## 1. Introduction

Rapid reduction of adult mosquito populations remains a central component of outbreak response to arboviral diseases such as dengue, chikungunya, and Zika. Because transmission intensity is directly linked to the abundance of adult female vectors, interventions targeting adults are widely prioritized during periods of elevated epidemiological risk [1,2,3]. In many tropical settings, chemical space spraying (commonly referred to as ‘fogging’) constitutes the primary adulticidal intervention deployed in response to increased vector abundance or reported cases [1,4,5]. Despite its widespread operational use, the long-term effectiveness, population consequences and economic implications of repeated fogging remain subjects of ongoing debate [6,7].

Space spraying involves dispersal of insecticidal aerosols into the air column to contact and kill flying adult mosquitoes. Two principal delivery approaches are employed. Thermal fogging vaporizes insecticide diluted in a carrier, typically diesel or kerosene, producing a visible fog. Ultra-low volume (ULV) cold fogging atomizes concentrated insecticide into fine droplets without thermal vaporization. Applications may be conducted using handheld equipment, backpack systems, or vehicle-mounted generators depending on scale and context [4,8]. In most operational programs, fogging is implemented as a discrete intervention and repeated at fixed intervals during outbreaks or high-transmission seasons [1].

The principal advantage of space spraying is its ability to produce rapid reductions in exposed adult mosquito populations. Because mortality occurs immediately upon contact with aerosol droplets, fogging can generate visible knockdown within hours. The method is operationally familiar to vector control programs and can be scaled across urban or resort environments. For these reasons, space spraying is widely regarded as an emergency response tool [1,4,8].

However, several limitations are well recognized. Mortality is restricted to mosquitoes present and contacted during application, while individuals resting indoors, within dense vegetation, or in cryptic habitats may escape exposure. Environmental conditions (e.g., wind) influence droplet dispersion and deposition [4]. Most importantly, space spraying does not directly affect immature stages; continued recruitment from larval habitats can result in rapid population rebound following application [5,6]. Sustained suppression therefore typically requires repeated treatments. Additionally, repeated reliance on chemical insecticides raises concerns regarding resistance development in mosquito populations [7]. These characteristics position fogging primarily as a short-term suppression intervention rather than a structural population management strategy [5,8].

In the Maldives, routine adult mosquito control on resort islands commonly includes handheld thermal fogging or misting, typically conducted on a weekly basis under normal operating conditions [9]. Applications are generally performed during pre-dawn or early evening hours using diesel- or kerosene-based thermal/ULV foggers operated by small field teams. However, spraying frequency may increase substantially during periods of elevated mosquito activity, such as the rainy season, or in response to guest complaints, in some cases occurring multiple times per week or even daily [10]. Given the geographically bounded nature of many resort islands and their strong emphasis on guest comfort, fogging is often implemented as a precautionary, service-oriented measure rather than strictly as an outbreak response. A review of practices on 37 resort islands (out of ~ 180 in operation to date) indicated that 26 (70.3%) spray at least once per day, and the rest at least once per week (Supplementary Figure S1). Despite its routine and sometimes intensified use, the long-term population effects of repeated high-frequency fogging in such settings have not been systematically quantified.

Mass trapping represents an alternative adult-control paradigm based on continuous removal rather than episodic mortality pulses [11]. Modern trapping systems typically combine visual cues, synthetic attractants, and carbon dioxide to target host-seeking female mosquitoes [12]. Deployed at defined densities across a treatment area, traps operate continuously and remove individuals throughout their activity cycles. Unlike fogging, which acts instantaneously and intermittently, trapping imposes sustained proportional removal over time.

The population impact of mass trapping depends strongly on deployment density relative to local recruitment rates. When removal (natural mortality + trapping) remains below recruitment (i.e., recruitment from immature stages within the island system), a reduced but stable equilibrium may be established. When removal exceeds recruitment, accelerated decline and potential collapse may occur [13]. Field evaluations have reported reductions in adult abundance under moderate to high trap densities in both urban and island settings [10,14,15]. In geographically bounded environments, such as islands, sustained high-density deployment has been associated with pronounced suppression and, in some cases, apparent local elimination of target species [13,16]. Nevertheless, outcomes are expected to vary depending on the target species, their behavioral ecology, immigration pressure, trapping efficiency and trap maintenance, and spatial coverage.

Mass trapping offers several potential advantages. Continuous removal does not depend on discrete application timing or specific meteorological windows. Reliance on broad-area insecticide dispersal is reduced, potentially contributing to resistance management strategies. Deployment density can be spatially optimized, allowing structured design of intervention layouts. At the same time, limitations must be acknowledged. Trapping typically requires higher initial capital investment than a single fogging round. Performance depends on consistent servicing, including lure replacement and consistent carbon dioxide supply. Effectiveness may decline in open systems with substantial immigration if trap density is insufficient to offset influx.

Space spraying disperses broad-spectrum insecticides into the environment and may expose non-target insects, particularly under repeated or high-frequency applications. Concerns have been raised regarding negative impacts on insect biodiversity, beneficial arthropods [17,18], aquatic life [19,20,21] and human health [22]. In contrast, attractant-based mass trapping targets host- or mate-seeking mosquitoes using species-specific cues and generally results in limited to no by-catch or environmental impact. These differences may influence the ecological footprint, environmental acceptability, and sustainability of adult control strategies.

Space spraying and mass trapping therefore represent fundamentally different operational paradigms for adult mosquito control: one characterized by episodic mortality pulses and rapid knockdown, the other by sustained proportional removal and density-dependent effects. While both aim to reduce adult vector abundance, their temporal dynamics, long-term population consequences, impact on non-target organisms and cost structures differ substantially.

Here, we compare space spraying and high-density mass trapping using a common population model parameterized with empirical adult mosquito data from our previous (island) studies [10,13,16], which targeted *Aedes aegypti* L. and *Ae. albopictus* Skuse. We hypothesize that space spraying produces rapid but transient reductions requiring repeated application to maintain suppression, whereas sufficiently dense mass trapping shifts equilibrium density and may induce sustained population collapse when removal exceeds intrinsic growth. By integrating mechanistic modeling with economic analysis, this study aims to inform strategic deployment decisions for evidence-based, environmentally sound and sustainable mosquito control on small islands, in the Maldives, but results are equally applicable to mosquito-infested physical or geographical islands elsewhere.

## 2. Materials and Methods

We compared space spraying (thermal or ULV fogging) and mass trapping (using Biogents AG mosquitaire traps and lures as described in detail by Jahir et al. [10]) as adult mosquito control strategies using a unified population-dynamics framework. The objective was to contrast episodic adulticide-induced mortality with continuous proportional removal under comparable biological assumptions.

The analytical approach integrated (i) mechanistic population modeling, (ii) numerical simulation of intervention scenarios, and (iii) bottom-up operational cost estimation.

### 2.1. Population-Dynamics Framework

Adult female mosquito dynamics were modeled as density-dependent recruitment balanced by mortality:

$$\frac{dN}{dt}=rN\left(1-\frac{N}{K}\right)-M\left(N\right)$$

where

*dN*/*dt* = rate of change in adult female mosquito density over time;

*N*(*t*) = adult female mosquito density at time *t*;

*r* = intrinsic per capita growth rate;

*K* = carrying capacity;

*M*(*N*) = intervention-induced mortality.

The logistic growth term represents reduced-form density dependence arising from larval habitat limitation and intraspecific competition. While mosquito life cycles are stage-structured, logistic formulations are widely used as parsimonious representations of net density regulation in vector population models [23,24,25]. This reduced-form approach captures the balance between recruitment and natural mortality without requiring explicit modeling of aquatic stages. The model assumes a geographically bounded system without immigration or emigration, such that population change arises solely from internal recruitment and mortality processes. Similar frameworks representing mosquito abundance as birth minus mortality processes have been used in dengue vector modeling [23,24], and density-dependent regulation has been empirically documented in mosquito populations [25]. The modeling framework was designed to compare generalized intervention types rather than evaluate specific products or brands.

### 2.2. Space Spraying

Space spraying was modeled as repeated pulse mortality applied at fixed intervals *T* (days). Let *N*_*i*_ denote adult density immediately after the *i*-th spray. Between sprays, the population follows logistic growth. Over one interval *T*, density immediately before the next spray is

$$N{(t}_{i+1}^{-})=\frac{K}{1+\left(\frac{K-N_{i}}{N_{i}}\right)e^{-rT}}$$

At time *t_i_*_+1_, proportional mortality is applied:

$$N_{i+1}=\left(1-\alpha \right)\frac{K}{1+\left(\frac{K-N_{i}}{N_{i}}\right)e^{-rT}}$$

where α is proportional mortality per application (0 < α < 1) and *T* is the spraying interval (e.g., *T* = 7 for weekly spraying). This links spray efficacy (α) and spray frequency (*T*) to long-term oscillatory dynamics. Fogging was assumed not to alter recruitment parameters (*r*, *K*), since spraying is known not to impact the aquatic population [5,6]. For each combination of α and *T*, long-term oscillatory mean density (${\bar{N}}_{\alpha }$) was computed numerically after transient dynamics had dissipated. Means were calculated over the final 30 days of a 120-day simulation.

### 2.3. Mass Trapping

Mass trapping was modeled as continuous proportional removal:

M(N)=ηcN

where

*c* = trap density (traps per hectare);

*η* = per-trap removal efficiency (per day).

The full system under trapping becomes

$$\frac{dN}{dt}=rN\left(1-\frac{N}{K}\right)-\eta cN$$

This formulation is mathematically equivalent to ‘harvesting’ models widely used in insect population dynamics and removal-trapping studies [26,27]. Continuous proportional removal from density-regulated populations has been extensively modeled for Dipteran vector systems, e.g., tsetse flies [28], and is directly applicable to mosquito trapping interventions.

Equilibrium density under trapping is

$$N^{*}=K\left(1-\frac{\eta c}{r}\right)$$

A critical trap density exists when

$$c_{crit}=\frac{r}{\eta }$$

For *c* > *c*_*crit*_, the only stable equilibrium is *N* = 0, resulting in sustained population collapse in geographically bounded systems (like islands).

### 2.4. Equivalence Between Trapping and Spraying

To directly compare interventions, we defined the equivalent trap density ($c_{eq}$) as the trap density that produces the same long-term mean density as repeated spraying:

$${\bar{N}}_{\alpha }=K(1-\frac{\eta c_{eq}}{r})$$

solving for $c_{eq}$,

$$c_{eq}=\frac{r}{\eta }\;(1-\frac{{\bar{N}}_{\alpha }}{K})$$

and thus to

$$c_{eq}=c_{crit}\;(1-\frac{{\bar{N}}_{\alpha }}{K})$$

This expression provides a direct analytical bridge between spray efficacy (α), spray interval (*T*), and the critical trap density ($c_{crit}$). Collapse-level equivalence ($c_{eq}\;$≥${\;c}_{crit}$) occurs only when ${\bar{N}}_{\alpha }$→ 0. This formulation allows translation of discrete spray-induced mortality into an equivalent continuous removal rate, enabling direct comparison of interventions with fundamentally different temporal structures.

### 2.5. Parameterization

Intrinsic growth rate (*r*) and equilibrium density (*K*) were estimated from empirical adult mosquito time-series data obtained from Maldivian island systems [13]. The empirical datasets used for parameterization were dominated by *Aedes aegypti* and *Ae. albopictus*, which represent the primary target species in the studied island systems. The trapping system used (Biogents mosquitaire trap) is specifically designed to attract host-seeking *Aedes* females, and model parameters therefore primarily reflect the population dynamics and trapping efficiency of these species. Removal efficiency (*η*) was derived from observed per-trap catch rates relative to estimated adult abundance [13]. Spray efficacy (*α*) was simulated across a conservative range (0.2–0.6), consistent with reported adulticide efficacy under field conditions [29,30,31]. Fogging frequency was set at daily (*T* = 1), twice weekly (*T* = 3.5) or weekly (*T* = 7), based on our survey of spraying practices in Maldivian resorts (Supplementary Figure S1). Estimates for trap densities at which removal exceeded recruitment followed outcomes of our previous studies [13]. Sensitivity analyses were conducted across biologically plausible ranges of *r*, *η*, and *α*.

### 2.6. Economic Analysis

A comparative economic evaluation of space spraying (thermal fogging or ULV fogging) and mass trapping was conducted from an operational provider perspective representative of Maldivian island resort settings. Deltamethrin is among the most widely used pyrethroid insecticides in mosquito control programs and is commonly applied in both ULV and thermal fogging operations, so this chemical was selected for our comparative cost analysis. Trap acquisition costs were included as capital expenditures and annualized over an assumed operational lifespan of 5 years using straight-line depreciation, thereby contributing to the per-hectare annual cost estimates for mass trapping. All costs were expressed in € per hectare per year (€ ha^−1^ year^−1^) to allow direct comparison across intervention intensities and frequencies (i.e., trap densities of 4, 8.6 or 12 ha^−1^, and spraying frequencies of once per week, twice per week or daily). A one-year analytic time horizon was used for annualized comparisons, with capital items amortized over their operational lifespan. No discounting was applied given the single-year primary analysis; however, capital depreciation was incorporated explicitly.

### 2.7. Use of AI

ChatGPT (OpenAI, version 5.2) was used to assist with model building and coding support for data analysis, as well as language editing and scientific phrasing. All analyses, outcomes and interpretations thereof and the final text were critically reviewed, verified, and approved by the authors, who take full responsibility for the content of this publication.

## 3. Results

### 3.1. Comparative Population Dynamics Under Space Spraying and Mass Trapping

**Figure 1:**
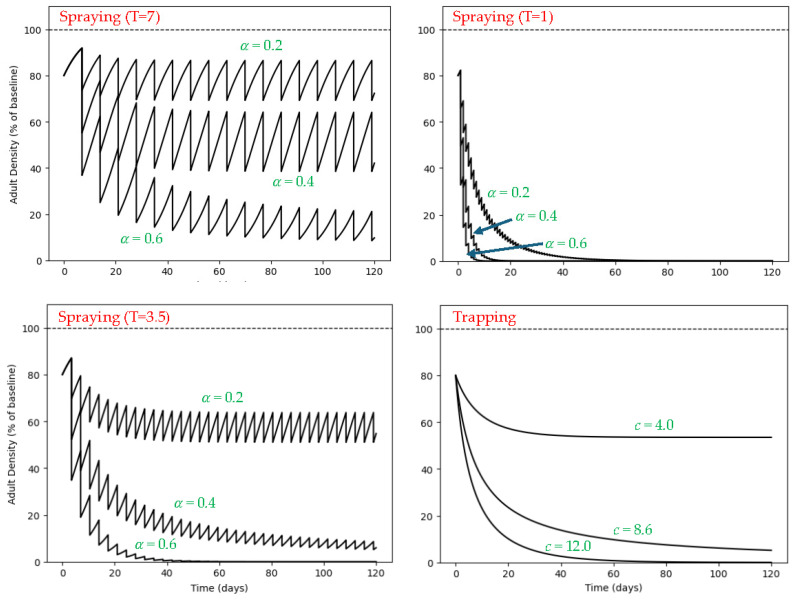
Population dynamics of adult female mosquitoes expressed as percentage of baseline (carrying capacity, 100%, dashed line) under space spraying and mass trapping in a closed system. **Top left**: weekly spraying (*T* = 7 days). **Bottom left**: twice-weekly spraying (*T* = 3.5 days). **Top right**: daily spraying (*T* = 1 day). Curves represent proportional mortality per application α = 0.2, 0.4, and 0.6. **Bottom right**: continuous mass trapping at densities c = 4.0, 8.6, and 12.0 traps ha^−1^.

The model reveals fundamentally different dynamical responses under space spraying and mass trapping (Figure 1). When expressed as percentage of baseline density (carrying capacity = 100%), weekly spraying (*T* = 7 days) produces persistent oscillatory dynamics whose amplitude depends on per-application mortality (α). Even at α = 0.6, density stabilizes at approximately 10–20% of baseline and does not collapse. Increasing spray frequency markedly amplifies suppression: twice-weekly spraying (*T* = 3.5) substantially lowers the long-term mean density, and daily spraying (*T* = 1) drives the system toward near-elimination even at moderate α values.

In contrast, mass trapping generates smooth convergence toward a new equilibrium determined by trap density (c). At c = 4 traps ha^−1^, density stabilizes at approximately 55% of baseline. In the baseline simulations [13], trapping at 8.6 traps ha^−1^ lay close to the transition between persistence and collapse, with trajectories approaching zero asymptotically. Higher densities produced more rapid decline. Unlike spraying, trapping alters the equilibrium structure of the system rather than producing periodic perturbations.

Direct comparison of curves across panels illustrates how combinations of α and *T* approximate specific trapping densities. For example, twice-weekly spraying with α = 0.2 stabilizes at roughly 55–60% of baseline, closely matching the equilibrium observed at c = 4 traps ha^−1^. Similarly, weekly spraying with α = 0.4 produces long-term densities near 45–50% of baseline, again comparable to outcomes slightly above c = 4. Weekly spraying with α = 0.6 converges toward ~15–20% of baseline, resembling the suppression achieved under trapping densities approaching, but still below, the critical threshold.

At higher frequencies, convergence becomes more pronounced. Twice-weekly spraying with α = 0.4 approaches densities below 10%, comparable to c ≈ 8.6 traps ha^−1^. Twice-weekly spraying with α = 0.6 and all daily spraying scenarios collapse the population toward zero, closely mirroring trapping outcomes at or above the critical density (c ≥ 8.6). Thus, specific α–*T* combinations generate suppression levels that visually and quantitatively correspond to distinct trapping densities.

Quantitative equivalence is summarized in Table 1. For fixed α values (0.2–0.6), the equivalent trap density (c_eq_) increases strongly with spray frequency. Under weekly spraying, α = 0.6 corresponds to c_eq_ ≈ 7.5 traps ha^−1^, remaining below the collapse threshold. Under twice-weekly spraying, α = 0.4 already approaches structural equivalence (c_eq_ ≈ 8.2 traps ha^−1^), and α = 0.6 exceeds it. Daily spraying produces near-complete suppression (~100%) even at α = 0.2, corresponding to c_eq_ ≈ 8.6 traps ha^−1^.

Together, these results demonstrate that spraying efficacy depends nonlinearly on the interaction between mortality per application (α) and frequency (T), whereas trapping efficacy depends primarily on whether trap density exceeds the model-estimated threshold density (8.6 traps ha^−1^).

### 3.2. Sensitivity to Intrinsic Growth Rate or Trapping Efficiency

Varying the intrinsic growth rate r altered recovery between spray events and therefore affected the magnitude of suppression achieved for a given α and T. Holding trap removal efficiency η constant, increasing r increased the implied critical trap density $c_{\mathrm{crit}}=r/\eta$, and reduced the percentage suppression achieved under fixed spraying scenarios. For weekly spraying, the equivalent trap density $c_{eq}$ changed only modestly with r at low mortality (α = 0.2) but became more sensitive at higher mortality (α = 0.6), reflecting faster rebound between spray pulses at higher r. Across schedules, shorter spray intervals (*T* = 3.5 and *T* = 1) reduced sensitivity to r, because limited recovery time between applications constrained rebound (Supplementary Table S1).

For mass trapping, the collapse threshold depends directly on the ratio r/η. Consequently, uncertainty in trap removal efficiency η translates into proportional shifts in the critical trap density $c_{\mathrm{crit}}$. Increasing η by 25% reduced $c_{\mathrm{crit}}$ by 20%, whereas decreasing η by 25% increased $c_{\mathrm{crit}}$ by 33% (Supplementary Table S2). This indicates that operational variation in trap performance (placement, maintenance, attractants, CO_2_ delivery, and environmental exposure) can materially shift the trap density required to cross the collapse threshold in bounded systems.

### 3.3. Time-to-Target Dynamics

Time-to-target (i.e., reducing the female mosquito population to 10% or 1% of its original size) analysis revealed contrasting operational profiles between spraying and trapping. Spraying produced rapid initial knockdown, reaching 50% of baseline within 3–7 days across scenarios. However, strong suppression (1% of baseline) required 45–50 days under weekly or twice-weekly spraying and 16 days under daily spraying. In contrast, trapping at densities below the critical threshold (c = 4 traps ha^−1^) did not reach 50% suppression. At the critical density (c = 8.6), decline was gradual, reaching 10% in approximately 58 days. At higher densities (c = 12), structural collapse occurred, with 10% suppression reached in ~20 days and 1% in ~56 days. These results highlight the distinction between rapid transient knockdown (spraying) and slower but structurally stable collapse (mass trapping).

### 3.4. Economic Comparison of Mass Trapping and Space Spraying

At a trap density of 4 traps ha^−1^, annual trapping costs were € 1621.36 ha^−1^ year^−1^, increasing to € 3485.92 ha^−1^ year^−1^ at the critical density (c_crit_ = 8.6 traps ha^−1^) and € 4864.08 ha^−1^ year^−1^ at 12 traps ha^−1^ (Supplementary Table S3). Lure (Mozzibait) replacement constituted the dominant cost component (69.2% of per-trap annual expenditure), followed by sugar for CO_2_ generation (10.2%) and labor (8.8%).

**Figure 2:**
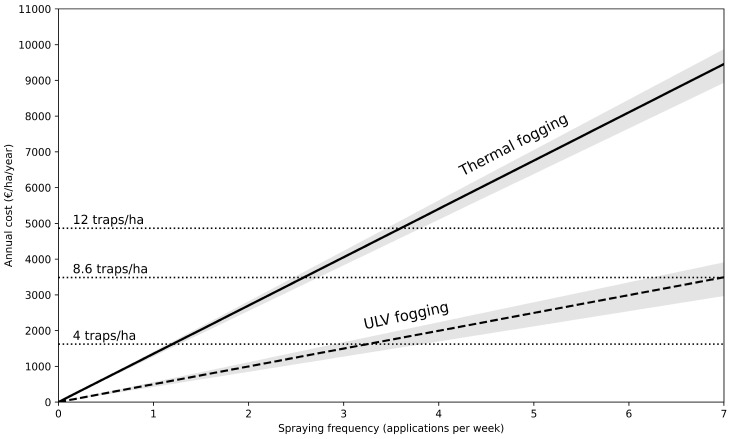
Annual control costs (€/ha/year) as a function of spraying frequency (applications per week), comparing thermal fogging (50 mL deltamethrin, 10 L diesel ha^−1^), ULV fogging (50 mL deltamethrin, 0.5 L diesel ha^−1^), and mass trapping at 4, 8.6 (c_crit_), and 12 traps ha^−1^. Thermal and ULV fogging costs increase linearly with application frequency, based on full operational costs of € 25.98 and € 9.59 per ha per application, respectively (Supplementary Table S4). Gray areas indicate how cost fluctuates if the price range for insecticide ranges from € 40 to € 85/L. Horizontal lines indicate annualized mass-trapping costs derived from Supplementary Table S3. Intersections represent break-even spraying frequencies at which fogging and trapping incur equivalent annual expenditure.

Thermal fogging (50 mL deltamethrin, 10 L diesel ha^−1^) incurred a full operational cost of € 25.98 ha^−1^ per application, equivalent to € 1350.96 ha^−1^ year^−1^ when applied weekly, € 2701.92 ha^−1^ year^−1^ when applied twice weekly, and € 9482.70 ha^−1^ year^−1^ under daily application (Supplementary Table S4). Diesel carrier represented the principal driver of thermal fogging costs (62.6% of total per-application expenditure). As shown in Figure 2, annual fogging costs increased linearly with spraying frequency. In contrast, ULV fogging (50 mL deltamethrin, 0.5 L diesel ha^−1^) substantially reduced per-application expenditure to € 9.59 ha^−1^, corresponding to € 498.68 ha^−1^ year^−1^ (weekly), € 997.36 ha^−1^ year^−1^ (twice weekly), and € 3500.35 ha^−1^ year^−1^ (daily) (Supplementary Table S4). In this scenario, insecticide, labor, and fogging equipment collectively accounted for a larger proportional share of total costs because carrier volume was much reduced because of the ultra-low volume (ULV) application.

Break-even frequencies differed markedly between thermal or ULV spraying. For thermal fogging, cost equivalence with trapping at c_crit_ (8.6 traps ha^−1^) occurred at approximately 2.6 spray applications per week, whereas equivalence with 4 traps ha^−1^ occurred at approximately 1.2 spray applications per week (Figure 2). At higher trap densities (12 traps ha^−1^), thermal fogging exceeded trapping costs when applied more frequently than approximately 3.6 times per week. Thus, with a large proportion of Maldivian resorts spraying at least once daily, trapping (especially at the recommended density of 10 traps ha^−1^) becomes economically competitive or favorable. ULV fogging, however, remained less expensive than trapping at 12 traps ha^−1^, and reached parity at 8.6 traps ha^−1^ under daily spray application. Cost equivalence with 4 traps ha^−1^ occurred at approximately three ULV spray applications per week. Thus, the economic competitiveness of spraying relative to trapping was highly sensitive to both application frequency and carrier volume.

## 4. Discussion

We provide, for the first time, a mechanistic comparison of episodic chemical space spraying and continuous proportional removal through mass trapping. By embedding both interventions within the same density-dependent population framework, we demonstrate that they differ not only operationally but structurally in their long-term population impact. Whereas space spraying (thermal or ULV) produces transient mortality pulses whose effectiveness depends on the interaction between per-application efficacy (α) and frequency (*T*), mass trapping alters the equilibrium structure of the system. Here, ‘structural suppression’ denotes a shift in the underlying population equilibrium toward a lower stable state or zero, rather than transient reductions around an unchanged equilibrium. When trap density exceeds a critical threshold (c_crit_ = 8.6 traps ha^−1^ in our parameterization, a value based on our analysis of multi-year trapping data from three islands in the Maldives and one island in the Philippines [13]), structural population collapse occurs. These contrasting dynamics have direct implications for operational strategy, sustainability, and policy design in geographically bounded systems such as small Maldivian islands.

A key point is that the threshold density identified here is not a universal empirical constant. Rather, it emerged from the specific parameterization of a closed, density-regulated system and therefore depends on intrinsic growth, trapping efficiency, and operational performance. The value of approximately 8.6 traps ha^−1^ used here should therefore be interpreted as a heuristic benchmark for comparable bounded island settings, not as a generally transferable cutoff. The island datasets analyzed here are consistent with this threshold-like behavior, but do not independently establish a statistically defined breakpoint. Nevertheless, based on prior work [13], these findings provide a pragmatic basis for selecting operational deployment densities, supporting the use of approximately 10 traps ha^−1^ as a robust and precautionary target for comparable bounded island settings.

### 4.1. Comparative Population Dynamics Under Space Spraying and Mass Trapping

The model confirms that spraying efficacy is nonlinear and frequency-dependent. Weekly spraying (even at high per-application mortality (α = 0.6)) does not induce collapse but produces persistent oscillatory dynamics around a reduced mean density. Increasing spray frequency progressively lowers the long-term mean, with daily spraying approaching elimination. This pattern reflects a simple biological mechanism: spraying does not alter recruitment parameters (r, K) since it does not affect the aquatic stages of the mosquito population. As long as recruitment exceeds removal between pulses, rebound occurs. Thus, spraying acts as a repeated perturbation of a structurally intact system.

In contrast, mass trapping imposes continuous proportional removal. Once ηc exceeds r, the equilibrium at N = 0 becomes globally stable. The system is no longer oscillatory but collapses deterministically toward extinction. This distinction (pulse perturbation versus structural threshold shift) represents the core mechanistic difference between the two strategies. Importantly, our equivalence analysis (Table 1) shows that certain α–*T* combinations approximate specific trapping densities. Our trapping data from Medhufaru island in the Maldives (49 ha in size, 4.1 trap ha^−1^, see [10,13]) yielded a stable equilibrium at 42.6% of baseline, which is better than our finding here (stable equilibrium at 55% of baseline). In other words, twice-weekly spraying (at a low proportion of mosquitoes killed per application), could be replaced with 4 traps ha^−1^, with the latter giving better outcomes. Nevertheless, only high-frequency spraying or high α values approach the 8.6 traps ha^−1^ threshold. Thus, routine weekly spraying (even if operationally common) rarely will achieve collapse-equivalent suppression. This likely explains why the frequency of spraying in many of the Maldivian resorts exceeds two rounds per week—a ‘better safe than sorry’ approach.

Empirical field evaluations of space spraying highlight substantial heterogeneity in adulticide effectiveness per application. In tropical urban settings such as Iquitos, Peru, operational ULV campaigns achieved measurable but transient reductions in adult *Aedes aegypti* abundance, consistent with per-application mortality estimates in the 30–50% range [32]. Thermal fogging trials in Martinique, including controlled sentinel cage exposures, have reported local knockdown as high as 80–90%, though field recovery often occurs within days and outcomes are severely compromised by insecticide resistance [33]. Similar moderate suppression (40–60%) was documented during outbreak responses in Brazil, with vector densities rebounding rapidly unless re-applications were frequent [34]. These empirical outcomes support our modeling choice of exploring α values between 0.2 and 0.6, capturing the range from conservative to strong but short-lived field efficacy.

### 4.2. Implications for Vector Control Strategies

Seventy percent of surveyed Maldivian resorts report spraying at least once per day (Supplementary Figure S1). This operational reality aligns with the model’s finding that high-frequency spraying is required to approach collapse-level suppression. However, this raises important strategic questions. First, daily spraying implies sustained chemical input that may accelerate resistance development and repeated operational costs. Daily or even weekly application of pyrethroids may result in strong resistance within 1–3 years [35]. Second, real-world α values may be lower than the conservative 0.2–0.6 range used in our simulations, particularly under suboptimal meteorological conditions or in structurally complex vegetation, like the dense jungle vegetation found on Maldivian islands (see Video S1 in Jahir et al. [10]). If α were <0.2 in practice, even daily spraying might fail to reach structural equivalence with c_crit_. The time-to-target analysis (Supplementary Figure S2) further highlights the distinction between rapid knockdown and durable collapse. Spraying achieves rapid initial reduction (50% within days), but strong suppression (to 1% of baseline) requires sustained high-frequency spraying. Trapping above the critical density shows slower initial decline but guarantees continued progression toward collapse without oscillatory rebound.

This has epidemiological implications. In outbreak response scenarios with a small infectious reservoir, rapid knockdown of infectious female mosquitoes may temporarily reduce transmission. However, if the human infectious reservoir remains substantial, rebound populations can quickly re-establish transmission potential [36]. Structural suppression strategies may therefore be more appropriate in geographically bounded systems where elimination or strong suppression is feasible.

### 4.3. Operational and Spatial Considerations

The sensitivity analyses (Tables S1 and S2) demonstrate that the conclusions are robust across plausible ranges of intrinsic growth rate (*r*) and trap efficiency (η). For spraying, increasing *r* accelerates rebound between pulses and reduces long-term suppression, particularly under weekly schedules. This suggests that high-productivity environments (e.g., during rainy seasons) require either increased α or shorter *T* to maintain suppression. For trapping, the collapse threshold scales linearly with *r* and inversely with η (c_crit_ = r/η). A ±25% variation in η shifts c_crit_ proportionally, emphasizing the importance of trap maintenance, lure integrity, CO_2_ delivery, and spatial placement. Nevertheless, even under conservative η values, collapse remains achievable at sufficiently high densities in bounded systems. From a spatial perspective, trapping offers design flexibility: density can be structured across interior grids or perimeter barriers [10,13,16]. Spraying, by contrast, is spatially diffuse and sensitive to wind and vegetation structure which may partly explain variability in field-observed α values [32,33,34]. The model assumes homogeneous spatial coverage of both spraying and trapping across the entire island. In practice, incomplete or uneven coverage would reduce effective intervention intensity, lowering realized spray efficacy (α) or the effective removal rate (ηc). As a result, insufficient spatial coverage may shift outcomes away from collapse and toward persistence, implying that the model results represent best-case scenarios under full deployment.

### 4.4. Economic, Ecological and Health Implications

The economic comparison between mass trapping and adulticide space spraying was highly sensitive to application frequency and, in the case of spraying, to carrier volume. Annualized costs derived from Supplementary Tables S3 and S4 and illustrated in Figure 2 show that both control strategies can shift from economically favorable to unfavorable depending on operational intensity. As such, thermal fogging remained less expensive than trapping at c_crit_ only when conducted fewer than approximately three times per week (Figure 2). Beyond this frequency, trapping became economically competitive or favorable. Diesel carrier was the dominant driver of thermal fogging expenditure (Supplementary Table S4) and reducing carrier volume to (ULV scenario) fundamentally altered this balance. ULV spraying was equally expensive as trapping at c_crit_ with biweekly lure replacement.

Taken together, these results highlight three economic determinants: (i) spraying frequency, (ii) carrier volume (and therefore fuel consumption), and (iii) lure replacement interval. From a programmatic perspective, the economic comparison is therefore not fixed but contingent on operational design parameters.

Importantly, the present analysis considers only direct operational expenditures. Several potentially substantial but difficult-to-quantify costs associated with space spraying were not included. Indiscriminate adulticide application affects non-target insect biodiversity, including pollinators and natural enemies, with possible cascading effects at higher trophic levels [17,18]. In island settings, repeated spraying may also contribute to contamination of surrounding marine ecosystems through drift and runoff, with unknown implications for coral reefs and coastal food webs [19]. In addition, potential impacts on human health (both occupational exposure of spray personnel and chronic low-level exposure of residents and guests) [22] were not monetized. Non-target capture in trapping systems was not quantified in this study. However, odor-baited traps designed for host-seeking mosquitoes are generally considered to have low by-catch relative to broad-spectrum insecticide applications. Nevertheless, species-specific differences in attraction and potential impacts on non-target insects warrant further empirical evaluation. Incorporating such ecological and public-health externalities would likely alter the relative cost-effectiveness comparison and could further shift the balance favorably toward mass trapping.

Although the present analysis indicates favorable outcomes for mass trapping under specific conditions, it is important to emphasize that the model structure itself is intervention-agnostic. The contrasting outcomes between space spraying and trapping arise directly from the mathematical representation of mortality as either episodic (pulse) or continuous (proportional), rather than from any intrinsic superiority of a specific commercial product or implementation. Any intervention capable of imposing sustained proportional removal exceeding intrinsic population growth would produce analogous threshold behavior, irrespective of the trapping technology used. Conversely, improvements in spray efficacy, coverage, or integration with larval control could substantially alter the relative performance of space spraying. The results presented here should therefore be interpreted as a comparison of control paradigms rather than specific products.

### 4.5. Limitations

First, the model assumes a closed system without immigration or emigration. While this assumption is appropriate for island settings, open urban systems with continuous mosquito influx may require substantially higher removal rates to achieve equivalent suppression. Incorporating dispersal dynamics would likely shift the critical control thresholds upward; immigration may partially offset removal and reduce the likelihood of achieving collapse-level suppression. Consequently, direct extrapolation of these results to non-island or open inland agroecosystems should be made with caution, as continuous immigration and spatial heterogeneity may alter intervention effectiveness. Second, insecticide resistance development was not modeled dynamically. This may lead to an overestimation of long-term spraying effectiveness under sustained operational use, particularly in settings with frequent application. Sustained chemical pressure can select for resistant phenotypes, effectively reducing spray efficacy (α) over time. As a result, long-term spraying effectiveness may decline unless resistance management strategies are implemented. The present model therefore represents short- to medium-term operational efficacy rather than evolutionary trajectories under prolonged selection. Third, the model adopts a reduced-form representation of density dependence and does not explicitly parameterize larval or pupal stages. As a result, interventions targeting immature stages, such as larval source management or larviciding, are not explicitly represented. Incorporating these processes would require a stage-structured modeling framework and may substantially alter recruitment dynamics and overall control outcomes. Fourth, as trap efficiency is species- and behavior-dependent, these findings are most directly applicable to *Aedes*-dominated systems and may not fully generalize to mosquito communities with different ecological or behavioral characteristics. Fifth, ecological externalities were not quantified. Space spraying may affect non-target arthropods, pollinators, and potentially adjacent marine systems in small island environments. Although these impacts are difficult to parameterize within a deterministic population model, they represent important external costs. Sixth, spraying and trapping were evaluated independently. In practice, hybrid strategies (such as initial rapid knockdown followed by sustained structural trapping) may offer synergistic advantages. Interventions within an integrated vector management (IVM) context were not simulated but warrant future modeling investigation. Of particular interest will be the integration of the Sterile Insect Technique with mass trapping, which we suspect to be a combination that will rapidly and sustainably eliminate mosquito populations from small islands.

## 5. Conclusions

Space spraying and mass trapping represent fundamentally different paradigms for adult mosquito control. Space spraying produces rapid but transient reductions in adult mosquito populations, with sustained suppression dependent on the interaction between per-application efficacy and application frequency. Mass trapping imposes continuous proportional removal and, under sufficiently high deployment densities in geographically bounded systems, can shift population dynamics toward sustained suppression or collapse. These contrasting dynamics suggest that the two approaches are not directly interchangeable, but rather operate on different temporal and mechanistic scales.

Space spraying remains an important tool for rapid response where immediate knockdown of adult populations is required. However, maintaining suppression through spraying alone may necessitate high-frequency application, with associated economic, operational, and resistance-related considerations. Mass trapping offers a pathway toward more stable, longer-term population reduction under conditions where sustained removal exceeds recruitment.

Importantly, the threshold-like behavior identified in this study arises from the specific parameterization of a closed, density-regulated system and should not be interpreted as a universal empirical constant. Instead, it provides a useful framework for understanding how intervention intensity relates to population outcomes and for guiding operational decision-making in comparable settings.

From a programmatic perspective, these findings support the view that integrated strategies combining rapid knockdown and sustained removal may offer a novel and effective approach to mosquito control. For example, initial space spraying to reduce high population densities could be followed by structured mass trapping to maintain suppression and prevent rebound. Future work should further refine estimates of spray efficacy under operational conditions, incorporate resistance dynamics, and explore the performance of combined intervention strategies within integrated vector management frameworks.

## Figures and Tables

**Table 1:** Equivalent trap density (c_eq_, traps ha^−1^) and long-term percentage suppression of adult mosquito density under repeated space spraying in a closed system. For fixed proportional mortality per application (α = 0.2, 0.4, 0.6), c_eq_ denotes the trap density that would produce the same long-term mean density as spraying applied at intervals *T* = 7 days (weekly), *T* = 3.5 days (twice weekly), or *T* = 1 day (daily). Percentage suppression is expressed relative to baseline carrying capacity (100%). Values approaching elimination are shown as ~100% to reflect asymptotic behavior.

α	Weekly c_eq_ (traps ha^−1^)	Weekly % Suppression	Twice-Weekly c_eq_ (traps ha^−1^)	Twice-Weekly % Suppression	Daily c_eq_ (traps ha^−1^)	Daily % Suppression
0.2	1.83	21.2%	3.66	42.5%	8.60	~100%
0.4	4.19	48.7%	8.15	94.8%	8.60	~100%
0.6	7.47	86.9%	8.60	~100%	8.60	~100%

## Data Availability

No new data were created or analyzed in this study. Data sharing is not applicable to this article.

## Supplementary Materials

The following supporting information can be downloaded at https://www.mdpi.com/article/10.3390/insects17050471/s1. Figure S1: Reported mosquito spraying frequency among Maldivian resorts (Tripadvisor-derived data); Figure S2. Time-to-target dynamics under space spraying and mass trapping; Table S1. Equivalent trap density (c_eq_) and long-term percentage suppression under repeated spraying (*T* in days) and spraying impacts (α) for varying intrinsic growth rate (r), with trap removal efficiency (η) held constant; Table S2. Trapping sensitivity showing critical trap density (c_crit_ = r/η) across r and η variation (±25% around baseline); Table S3. Mass-trapping costs per trap (€/trap/year; top or €/ha/year; bottom) and annualized equivalents; Table S4. Full operational fogging (thermal or ULV) costs per hectare per application (€/ha/application) and annualized equivalents.
