# Supplementary material for: From Transient Knockdown to Density-Driven Collapse: A Mechanistic Comparison of Adult Mosquito Control by Space Spraying and Mass Trapping in Maldivian Islands
Source: Insects. 2026 May 2;17(5):471. doi: 10.3390/insects17050471 (PMC13207721; doi:10.3390/insects17050471)
Supplement: Supplementary file 1 [file insects-17-00471-s001.zip › Figure S1.pdf]

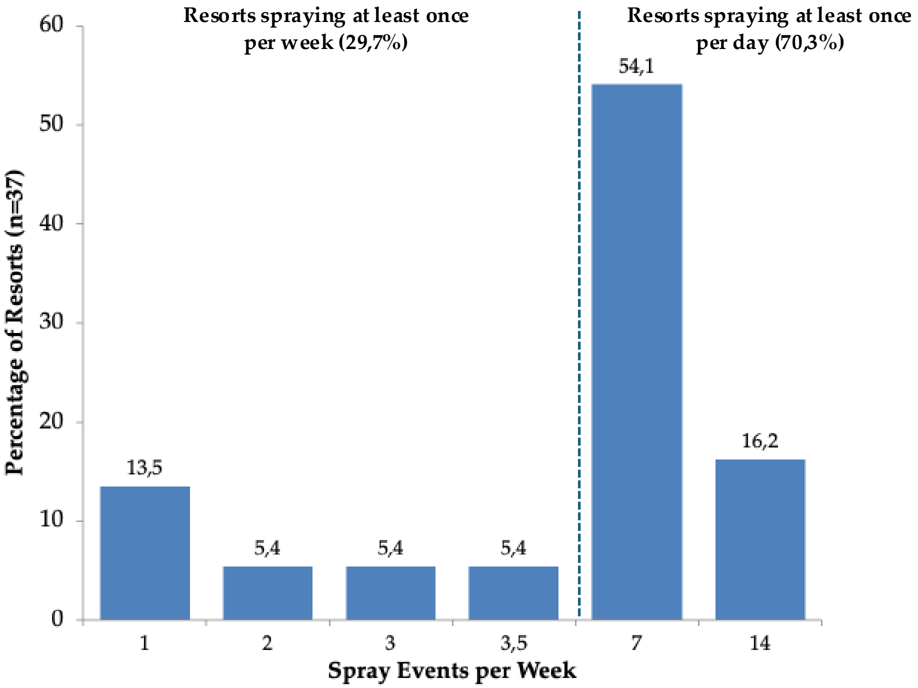

Figure S1. Reported mosquito spraying frequency among Maldivian resorts (Tripadvisor-derived data).

Bar graph showing the percentage of resorts grouped by reported mosquito control spraying frequency. A total of 100 Maldivian resorts were screened, representing approximately 55% of the ~180 operational resort islands in the Maldives as of 2025 (Maldives Tourism Yearbook).

Of the 100 resorts examined, 37 contained explicit statements specifying a spraying frequency and were included in the analysis; the remaining resorts either had no mention of spraying or used non-quantified terms (e.g., 'regular fogging') and were excluded. Thus, explicit spraying frequency information was available for approximately 21% of all operational Maldivian resort islands nationwide (37 of ~180).

For each resort, publicly available Tripadvisor reviews and Q&A content were screened using the keywords fogging, misting, spraying, or fumigation. Only statements explicitly mentioning a frequency (e.g., 'twice a day,' 'once a week,' etc.) were included. Each resort was classified according to the clearest reported frequency based on the extracted review text. The dataset reflects reviewer-reported operational practices at the time of posting and does not constitute independently verified resort policy. Also, seasonal variation in spraying frequency was not taken into account.
