# Supplementary material for: From Transient Knockdown to Density-Driven Collapse: A Mechanistic Comparison of Adult Mosquito Control by Space Spraying and Mass Trapping in Maldivian Islands
Source: Insects. 2026 May 2;17(5):471. doi: 10.3390/insects17050471 (PMC13207721; doi:10.3390/insects17050471)
Supplement: Supplementary file 1 [file insects-17-00471-s001.zip › Figure S2.pdf]

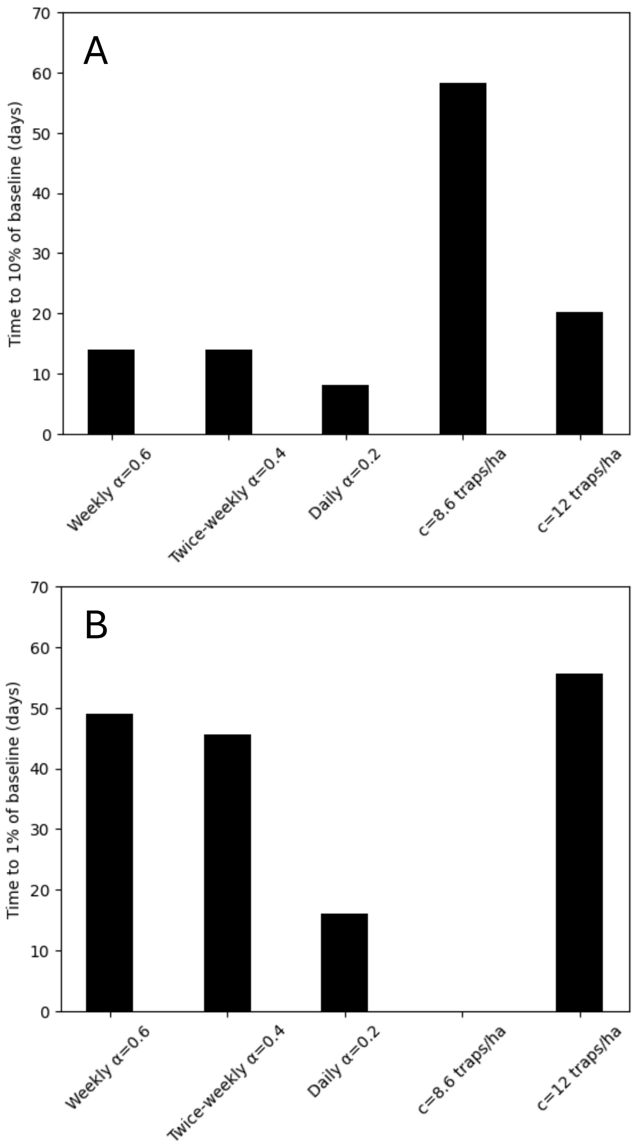

Figure S2. Time-to-target dynamics under space spraying and mass trapping. (A) Time required to reduce adult mosquito density to 10% of baseline carrying capacity (K). (B) Time required to reduce density to 1% of baseline. Spraying scenarios represent repeated pulse mortality with weekly (α = 0.6), twice-weekly (α = 0.4), and daily (α = 0.2) applications. Trapping scenarios represent continuous proportional removal at densities of 8.6 traps ha^{-1} (critical density) and 12 traps ha^{-1} (above threshold). Spraying produces rapid initial knockdown but requires sustained frequency to achieve deep suppression. In contrast, trapping above the critical density produces structural population collapse, with slower but stable progression toward low-density states.
