# Supplementary material for: From Transient Knockdown to Density-Driven Collapse: A Mechanistic Comparison of Adult Mosquito Control by Space Spraying and Mass Trapping in Maldivian Islands
Source: Insects. 2026 May 2;17(5):471. doi: 10.3390/insects17050471 (PMC13207721; doi:10.3390/insects17050471)
Supplement: Supplementary file 1 [file insects-17-00471-s001.zip › Table S1.pdf]

Table S1. Equivalent trap density (c_{eq}) and long-term percentage suppression under repeated spraying (*T* in days) and spraying impacts (α) for varying intrinsic growth rate (r), with trap removal efficiency (η) held constant.

Spray schedule (in days, *T*)	α	r (day^{-1})	Implied $c_{crit}= r/\eta$ (traps ha^{-1})	c_{eq} (traps ha^{-1})	% suppression
7	0.2	0.1	5.73	1.81	31.6
7	0.2	0.15	8.6	1.8	21.0
7	0.2	0.2	11.47	1.79	15.6
7	0.4	0.1	5.73	4.16	72.6
7	0.4	0.15	8.6	4.15	48.2
7	0.4	0.2	11.47	4.13	36.0
7	0.6	0.1	5.73	7.42	95.5
7	0.6	0.15	8.6	7.47	86.9
7	0.6	0.2	11.47	7.52	77.8
3.5	0.2	0.1	5.73	3.6	56.8
3.5	0.2	0.15	8.6	3.66	42.5
3.5	0.2	0.2	11.47	3.71	33.7
3.5	0.4	0.1	5.73	7.36	93.9
3.5	0.4	0.15	8.6	8.15	94.8
3.5	0.4	0.2	11.47	8.74	86.2
3.5	0.6	0.1	5.73	8.6	~100
3.5	0.6	0.15	8.6	8.6	~100
3.5	0.6	0.2	11.47	8.6	~100
1	0.2	0.1	5.73	8.6	~100
1	0.2	0.15	8.6	8.6	~100
1	0.2	0.2	11.47	8.6	~100
1	0.4	0.1	5.73	8.6	~100
1	0.4	0.15	8.6	8.6	~100
1	0.4	0.2	11.47	8.6	~100
1	0.6	0.1	5.73	8.6	~100
1	0.6	0.15	8.6	8.6	~100
1	0.6	0.2	11.47	8.6	~100
