# Supplementary material for: From Transient Knockdown to Density-Driven Collapse: A Mechanistic Comparison of Adult Mosquito Control by Space Spraying and Mass Trapping in Maldivian Islands
Source: Insects. 2026 May 2;17(5):471. doi: 10.3390/insects17050471 (PMC13207721; doi:10.3390/insects17050471)
Supplement: Supplementary file 1 [file insects-17-00471-s001.zip › Table S2.pdf]

Table S2. Trapping sensitivity showing critical trap density ($c_{crit}=r/\eta$) across r and η variation (±25% around baseline).

η multiplier	η (day^{-1} per trap)	r (day^{-1})	$c_{crit}=r/\eta$ (traps ha^{-1})
0.75	0.01308	0.1	7.64
0.75	0.01308	0.15	11.47
0.75	0.01308	0.2	15.29
1.0	0.01744	0.1	5.73
1.0	0.01744	0.15	8.6
1.0	0.01744	0.2	11.47
1.25	0.0218	0.1	4.59
1.25	0.0218	0.15	6.88
1.25	0.0218	0.2	9.17
