# Supplementary material for: From Transient Knockdown to Density-Driven Collapse: A Mechanistic Comparison of Adult Mosquito Control by Space Spraying and Mass Trapping in Maldivian Islands
Source: Insects. 2026 May 2;17(5):471. doi: 10.3390/insects17050471 (PMC13207721; doi:10.3390/insects17050471)
Supplement: Supplementary file 1 [file insects-17-00471-s001.zip › Table S3.pdf]

Table S3. Mass-trapping costs per trap (€/trap/year; top or €/ha/year; bottom) and annualized equivalents. Based on a 5-year trap lifespan (€ 95.50 per trap + CO_2 release nozzle), biweekly lure (Mozzibait, Biogents AG, Regensburg, Germany) replacement (€ 10.78 per sachet; 26 replacements per year), and CO_2 generation via yeast–sugar fermentation (20 g yeast and 700 g sugar per trap every three days). Annual consumables per trap were 2.43 kg yeast and 85.2 kg sugar, costed using 2026 procurement prices (US$ 2.86 per 500 g yeast; US$ 0.51 per kg sugar) and converted to € at the exchange rate 1 US$ = 0.951 €. Traps operate on 12V DC (3.6 W), which on Maldivian islands that mostly generate power through diesel generators (at an average of 40% efficiency conversion of 10 kWh/L), results in € 11,80 per trap per year. Servicing was conducted by a two-person team earning US$ 375.00 per person per month, maintaining 80 traps per day on a three-day servicing cycle (240 traps supported continuously). Transport costs were based on a utility buggy costing US$ 2,000 with a 2-year lifespan. Capital items were annualized linearly over their operational lifespan. Percentages indicate the proportional contribution of each cost component to total annual per-trap expenditure.

Item	Cost per unit	Cost per trap per year (€)	% Of total
Trap + Nozzle (CO_2)	€ 95.50 (5-year life)	19.10	4.71
Lure (Mozzibait)	€ 10.78 per sachet (26/year)	280.28	69.15
Yeast (CO_2)	US$ 5.72/kg (5.44 €/kg)	13.22	3.26
Sugar (CO_2)	US$ 0.51/kg (0.49 €/kg)	41.32	10.19
Labor (2 staff)	US$ 375.00/month/person	35.66	8.80
Power supply	€ 11.80/trap/year	11.80	2.91
Buggy (transport)	US$ 2,000.00 (2-year life)	3.96	0.98
TOTAL		405.34	100.0

Annualized trapping costs (€/ha/year)

Trap density (c, number ha^{-1})	Cost per hectare per year (€)
4	1,621.36
8.6 (= c_{crit})	3,485.92
12	4,864.08
