# Supplementary material for: From Transient Knockdown to Density-Driven Collapse: A Mechanistic Comparison of Adult Mosquito Control by Space Spraying and Mass Trapping in Maldivian Islands
Source: Insects. 2026 May 2;17(5):471. doi: 10.3390/insects17050471 (PMC13207721; doi:10.3390/insects17050471)
Supplement: Supplementary file 1 [file insects-17-00471-s001.zip › Table_S4.pdf]

Table S4. Full operational fogging (thermal or ULV) costs per hectare per application (€/ha/application) and annualized equivalents. Costing is for a two-person fogging team using deltamethrin (1.25%) at 50 mL/ha with 10 L or 0.5 L diesel/ha as carrier for thermal and ULV fogging, respectively. Labour was costed at US$ 375 per person per month and converted at 1 US$ = 0.951 €. Productivity was assumed at 3 ha h^{-1} with 20% non-productive time (mixing, refilling, relocation), giving 2.5 ha h^{-1} effective output. A standard 176 h $month^{-1}$ was used to derive an hourly labour rate. Fogger running costs (fuel + maintenance + depreciation) were set to € 4.30 h^{-1}. PPE (gloves, boots, mask, clothing)/admin consumables were € 1.00 ha^{-1} per application. Overheads/management were applied as 15% of direct costs.

Item	Cost per unit	Thermal fogging cost (in €) per ha per application (%)	ULV fogging cost (in €) per ha per application (%)
Deltamethrin (1.25%)	€ 65.00 L^{-1}; 50 mL ha^{-1}	3.25 (12.51)	3.25 (33.89)
Diesel carrier	€ 1.50 L^{-1}; 10 L ha^{-1} (thermal) or 0.5 L ha^{-1} (ULV)	15.00 (57.73)	0.75 (7.82)
Labor (2 staff)	US$ 375 $month^{-1}$ $person^{-1}$ (converted to €)	1.62 (6.23)	1.62 (16.90)
Thermal fogger (maintenance + depreciation)	€ 4.30 h^{-1}; 2.5 ha h^{-1} effective	1.72 (6.62)	1.72 (17.94)
PPE / admin consumables	€ 1.00 ha^{-1} $application^{-1}$	1.00 (3.85)	1.00 (10.43)
Overhead / management	15% of direct costs	3.39 (13.06)	1.25 (13.03)
TOTAL		25.98 (100)	9.59 (100)

Annualized spraying costs (€/ha/year)

Spraying frequency	Applications per year	Thermal fogging cost per ha per year (€)	ULV fogging cost per ha per year (€)
Weekly	52	1,350.96	498.68
Twice weekly	104	2,701.92	997.36
Daily	365	9,482.70	3,500.35
